# Contribution of universities health and safety services in achieving the sustainable development goals

**DOI:** 10.3389/fpubh.2025.1604003

**Published:** 2025-07-29

**Authors:** M. Samper Rivas, C. Salcines, P. López-Roldán, S. Rosa, D. Monge Martín, E. Colino

**Affiliations:** ^1^Safety, Health and Welfare Service, Universidad Francisco de Vitoria, Madrid, Spain; ^2^University of Cantabria, Health and Safety Unit, Infrastructure Service, Santander, Spain; ^3^Occupational Risk Prevention Service, Universidad de Córdoba, Córdoba, Spain; ^4^Human Resources & Occupational Safety and Health Services, Universidad Carlos III de Madrid, Madrid, Spain; ^5^Faculty of Medicine, Universidad Francisco de Vitoria, Madrid, Spain; ^6^Faculty of Health Sciences, Universidad Francisco de Vitoria, Madrid, Spain

**Keywords:** higher education, sustainability, sustainable development goals, Occupational Health & Safety, university

## Abstract

**Introduction:**

Universities play a pivotal role in advancing the Sustainable Development Goals (SDGs) and the 2030 Agenda through their educational mission, research activities, and contributions to social well-being, economic progress, and social cohesion. Beyond their conventional functions, universities can contribute to sustainability through the specific actions of their Occupational Health and Safety (OHS) services. This study explores the contribution of university OHS services to the achievement of the SDGs within the Spanish higher education system.

**Methods:**

A cross-sectional survey was conducted among Spanish universities to identify OHS actions linked to the SDGs and to assess their institutional impact. The study presents a structured methodology that classifies OHS actions into specific and general categories, mapped across three strategic dimensions: person, environment, and culture.

**Results:**

Results reveal that OHS services contribute to a wide range of SDGs, extending beyond the traditionally recognized SDG 3 (Good Health and Well-being) and SDG 8 (Decent Work and Economic Growth).

**Discussion:**

This research provides an original SDG Impact Matrix that highlights the multidimensional role of OHS services in fostering sustainability within universities. The findings offer valuable insights for integrating OHS strategies into institutional sustainability policies and expanding their role as active agents in the advancement of the 2030 Agenda.

## 1 Introduction

The Sustainable Development Goals (SDGs) are a universal call. Acting to end poverty, protect the environment and improve the lives and futures of people around the world; a blueprint for a sustainable future for all. In 2015, the General Assembly of the United Nations (UN) adopted a resolution in which all Member States agreed to 17 SDGs as part of the 2030 Agenda for Sustainable Development (1).

This resolution was followed by a significant response from most Member States, including governments (2), businesses and organizations (3–7). Universities, as essential agents of economic growth, innovation and social progress, have been no exception in this sense, and have contributed in a number of important ways to meet the challenges of the SDGs (8–18).

Several international studies have highlighted the role of universities as key agents in the implementation of the SDGs, not only through their educational and research functions but also through their internal policies and governance structures. In Scandinavia, for example, comprehensive sustainability practices have been documented in universities in Sweden and Norway, with a strong emphasis on institutional participation (19). In a global study, Lozano et al. (20) demonstrated how institutional commitment to sustainable development has significantly evolved in universities across all continents, highlighting progress in governance, teaching, research, and community engagement.

These international experiences reinforce the relevance of the present study and support its approach as a replicable and adaptable contribution for other university contexts aiming to advance the transversal implementation of the SDGs.

In Spain, Crue Spanish Universities –a non-profit association that groups a total of 76 Spanish universities, public and private– has demonstrated its commitment to the 2030 Agenda and the SDGs. In 2019, Crue set up a commission to coordinate joint actions by universities to achieve these goals and promote environmental awareness within the university community. This commission encouraged Spanish universities to embrace the 2030 Agenda, deploying their resources, capabilities, and influence to help achieve the SDGs (4).

According to the International Labor Organization (ILO), around 6,400 people worldwide die from work-related accidents or illness every day, totaling 2.3 million deaths per year, while an additional 860,000 people suffer on-the-job injuries every day. Urgent action is therefore necessary to create a global culture of health and safety in the workplace that guarantees workers’ rights to a safe and healthy work environment while ensuring that both employees and employers know their rights and their responsibilities. Among all the 17 SDGs, the Occupational Health and Safety (OHS) initiatives of the ILO focus on SDG 3 –“Good Health and Well-being”– and SDG 8 –“Decent Work and Economic Growth”–. Regarding SDG 3, the ILO emphasizes that a safe and healthy work environment is essential to both environmental sustainability and greater productivity. Workplace accidents and occupational illnesses are a severe hindrance to achieving this SDG. Regarding SDG 8, the ILO believes it is essential to establish fundamental labor rights and standards on a national level. Thus, the OHS community must take a comprehensive approach in addressing the profound changes facing the world of work in order to achieve SDG 8.

At a European level, the European Health Union is currently being developed in the belief that the right to a safe and healthy workplace is fundamental in achieving the SDGs. In fact, the European Commission is working with the ILO and the World Health Organization (WHO) sharing data and expertise and working in partnership with Member States to develop a new indicator of workplace-related mortality due to occupational illnesses and other associated risks. This cooperative initiative clearly shows that the European Union (EU), the ILO and the European workers all share the same goals, that is, decent work for all in a better society, in line with Europe’s SDGs.

There is a close link between OHS and sustainability. Organizations committed to providing a safe and healthy work environment are more likely to be more successful in achieving the SDGs. Built-in safety design, risk assessment and OHS management are all essential to long-term business sustainability.

Given the urgency of addressing the significant challenges in terms of health, safety and well-being as established in the SDGs, organizations play a fundamental role in ensuring an optimal quality of life given the direct and indirect impact of their actions on individuals. Therefore, the challenge of sustainability must be taken up not only by governments and nongovernmental organizations, but also by companies and other institutions, including universities.

In any organization, the departments responsible for ensuring the health and safety of workers play a fundamental role in achieving SDG 3 and SDG 8 as ILO highlighted.

The point of departure for this study was the 2018 SDG project by the Crue Sustainability Area with the purpose of identifying and aligning Crue’s lines of work with the SDG objectives. To this end, a survey was conducted in 2019 to identify and raise awareness of the actions, commitments and alliances of universities as part of the effort to achieve the SDGs.

The results of the survey revealed that the area of OHS linked to 6 specific SDGs. In other words, within the Spanish university system, the actions of the OHS Services have an impact on far more SDGs than those initially signaled by the ILO.

The purpose of the study was to identify the impact of the activities carried out by the OHS Services of Spanish universities on achieving the SDGs and their contribution of value to society.

## 2 Materials and methods

### 2.1 Study design

To identify the activities of the OHS Services linked to the SDGs, a cross-sectional observational study was carried out to analyze the distribution of different variables during a given period.

### 2.2 Settings and participants

The study included all OHS Services of Spanish universities associated with Crue. These services, essential in any organization (21, 22), are particularly important at universities by contributing to fostering a culture of health and safety in education, research and innovation and changing attitudes and behaviors throughout society (4, 10, 22). Within the 2020–2021 academic year, the Spanish University System was composed of 84 universities −50 public, 34 private–. At the time of the study, 76 universities (90.48%) were associated with Crue −50 public, 26 private–.

The target population of the study was the OHS services subscribed to the mailing list of the Working Group on OHS of the Crue-Sustainability Area Commission. At the time of the study, this list included 60 universities with 261 registered email addresses; that is, 71.43% of all universities in Spain and 78.95% of the universities associated with Crue.

Participation in the study was voluntary. Although this approach may introduce a certain degree of selection bias, favouring the OHS Services of universities with a stronger commitment to sustainability and the SDGs, it also enables the identification of good practices and advanced approaches that may serve as valuable references for other institutions.

The survey was distributed twice (September 2020 and February 2021) to recruit additional participating universities and increase the number of responses. Prior to the beginning of the survey, an informative session was incorporated. The survey remained open for a month on both occasions, with a channel provided to answer any questions.

The participation rate was 48.33% of the target population (29/60 universities), which reflects a balanced composition in terms of institutional type (public/private), territorial distribution, and university size, providing heterogeneity and robustness to the results. Although no formal non-response bias analysis was conducted, the qualitative richness and the degree of specificity of the reported actions reinforce the validity of the study as a first empirical approach to the link between OHS services and the SDGs within the Spanish university system.

**Figure 1:**
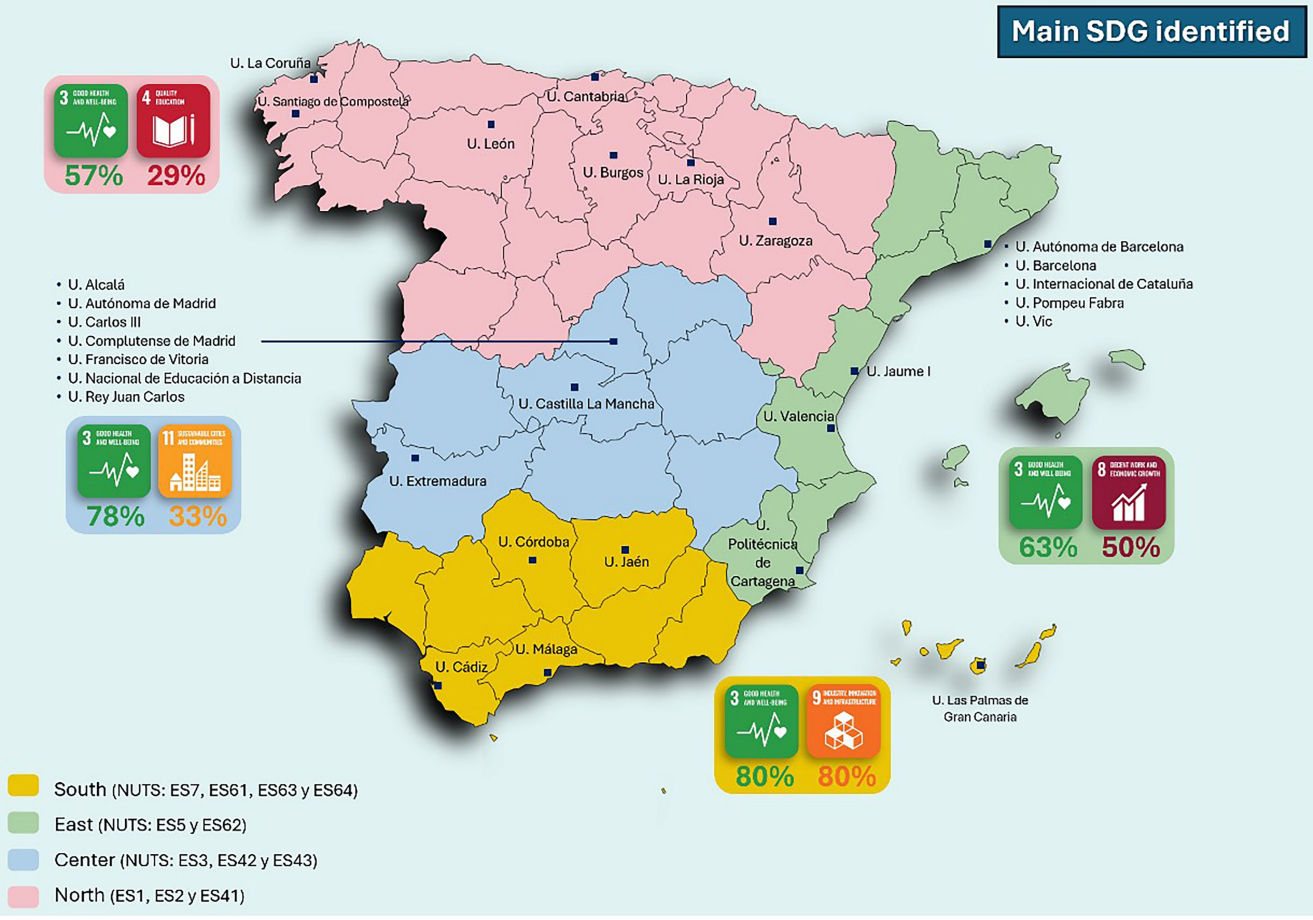
Geographical distribution of participating universities.

Regarding the territorial impact of the survey, it is worth highlighting that universities from all regions of Spain participated, as illustrated in Figure 1. The regional classification used in this study was based on geographic proximity and spatial distribution within Spain, following the Nomenclature of Territorial Units for Statistics (NUTS). Both NUTS 1 (major socioeconomic regions) and NUTS 2 (basic statistical regions) were considered. Specifically, the ‘North’ region comprises NUTS 1 zones ES1 (Northwest) and ES2 (Northeast), and NUTS 2 region ES41 (Castile and León). The ‘Central’ region includes NUTS 1 zone ES3 (Community of Madrid) and NUTS 2 regions ES42 (Castile-La Mancha) and ES43 (Extremadura). The ‘South and Canary Islands’ region comprises NUTS 1 zone ES7 and NUTS 2 regions ES61 (Andalusia), ES63 (Ceuta), and ES64 (Melilla). Finally, the ‘East’ region corresponds to NUTS 1 zone ES5 and NUTS 2 region ES62 (Region of Murcia).

The number of responses was higher in the central and northern areas of the country, particularly in the Autonomous Communities of Madrid and Catalonia, which concentrate a greater number of universities compared to other regions, with 14 and 12 universities, respectively. By region, universities in the North primarily identified SDG 3 (57%, 4 out of 7 participating universities) and SDG 4 (29%, 2 out of 7), the latter with results comparable to other identified SDGs. In the Central region, SDG 3 (78%, 7 out of 9) and SDG 8 (33%, 3 out of 9) were the most frequently cited. In the Levante region, SDG 3 was identified by 63% of universities (5 out of 8), followed by SDG 8, identified by 50% (4 out of 8). Finally, in the Southern region and the Canary Islands, SDGs 3 and 9 were predominant, both being identified by 80% (4 out of 5) of the universities in that area.

Regarding the type of institution, 26 public universities participated, representing 52% of all public universities in Spain, and 3 private universities participated, accounting for 11.54% of all Spanish private universities.

### 2.3 Variables and measurements

The data was obtained through an online questionnaire consisting of 9 questions elaborated by representatives of the SDG Safety and Health Project initiated by the OHS working group of the Crue-Sustainability Area Commission.

The questionnaire was developed by professionals with extensive experience in the field of Occupational Health and Safety, all of whom were members of the OHS Working Group of Crue. Although the instrument was peer-reviewed and pre-tested during an informational session to ensure its clarity, consistency, and relevance prior to its final implementation, no formal construct validation or reliability testing (e.g., internal consistency) was conducted. This decision reflects the descriptive and exploratory design of the study, which aimed to gather institutional practices rather than measure latent constructs. As a result, the findings should be interpreted with this limitation in mind, particularly in terms of generalizability and the potential variability in how respondents may have understood or categorized their institutional actions.

Data analysis focussed particularly on 4 questions referring specifically to the impact of the OHS Services on achieving the SDGs, their contribution to the university environment and the degree of participation of OHS personnel in the sustainability working groups at the university. These four questions (questions 3, 4, 5 and 7 of the questionnaire) were as follows:

*Q3*: Does the OHS Service participate in specific working groups related to the SDGs of the 2030 Agenda at the University? Closed question, answer Yes/NO.
*Q4*: Do officer or medical staff of the OHS Service participate in these working groups? Closed question, answer Yes/No.
*Q5*: In your university, which SDGs do you identify as OHS actions or initiatives? This is a multiple-choice question, where you select the SDG(s) that identifies the OHS actions.
*Q7*: Do you think that the preventive activities developed by the OHS Services could contribute to achieving the SDGs? Closed question, answer Yes/No.

The rest of the questions were aimed at obtaining specific information on each university’s OHS initiatives that had an impact on the SDGs and on the proposals that the universities themselves could make on this issue. Thus, question 6, an open-ended question, collected the actions or OHS initiatives developed by each university, according to the SDGs on which they had an impact. Question 8, also an open-ended question, had the objective of asking each university to make proposals for OHS initiatives to achieve the SDGs in the Spanish university environment. Finally, questions 1, 2 and 9 aimed to identify the participating university and to report on the protection of personal data. The complete questionnaire is provided as a Supplementary file.

### 2.4 Statistical analysis

A descriptive statistical analysis was carried out to assess the characteristics of the sample. Qualitative variables were presented as absolute and relative frequencies (percentages). For quantitative variables, measures of central tendency (mean, median) and dispersion (standard deviation, interquartile range) were calculated. All analyses were performed using SPSS version 25 statistical software (IBM Corp.)

## 3 Results

### 3.1 Phase 1: specific actions

**Figure 2:**
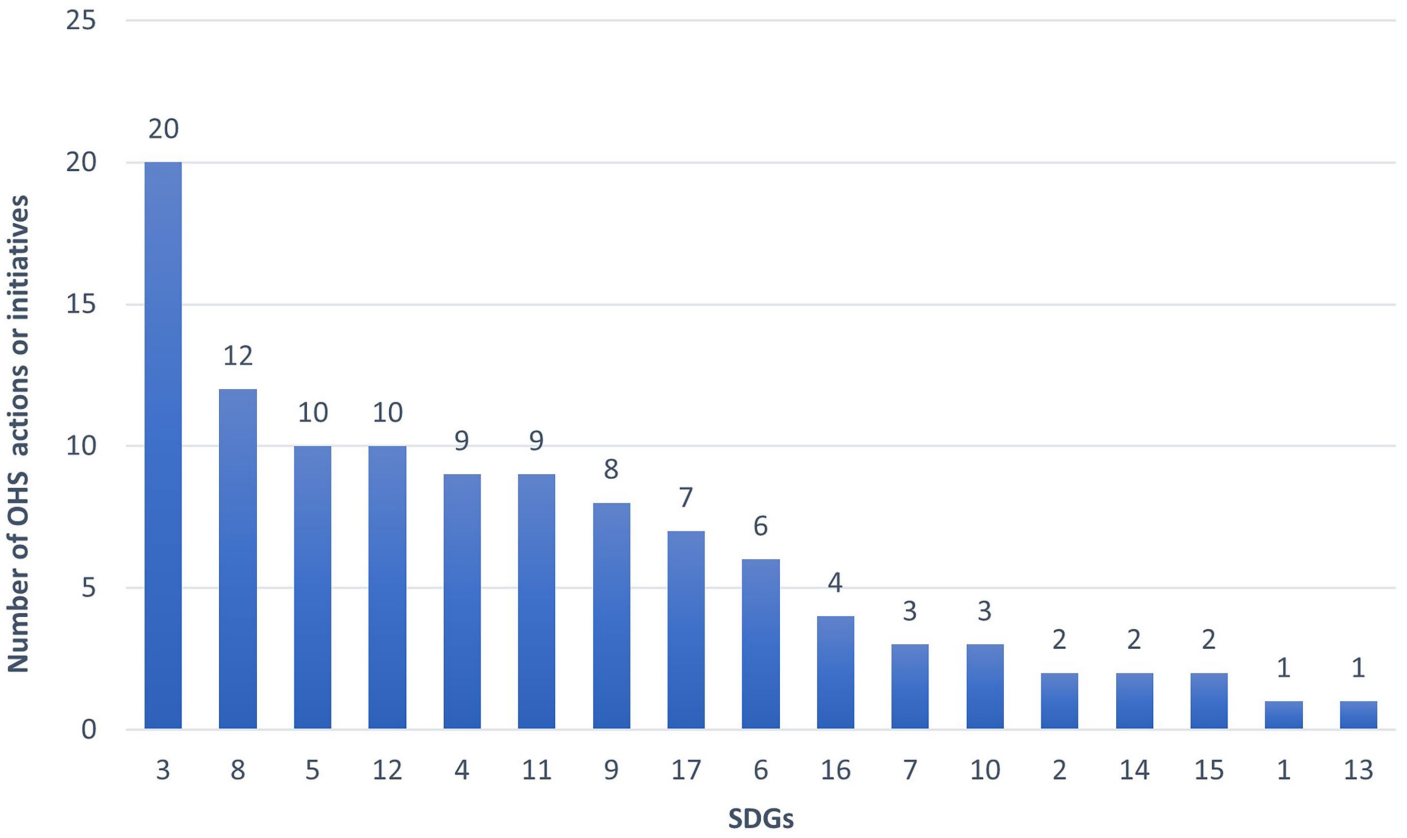
Number of SDGs associated with occupational health and safety (OHS) actions or initiatives.

Figure 2 shows the number of SDGs associated with the OHS actions identified by the participating universities. Notably, SDG 3 (Good Health and Well-being) and SDG 8 (Decent Work and Economic Growth) are the most frequently cited, which aligns with the traditional link between OHS and these goals. However, it is particularly relevant that other SDGs, such as SDG 5 (Gender Equality), SDG 12 (Responsible Consumption and Production), SDG 4 (Quality Education), and SDG 11 (Sustainable Cities and Communities), also emerge as areas where OHS services have a tangible impact. This finding underscores the multidimensional contribution of OHS services beyond conventional occupational risk prevention.

All participants believe the actions of the OHS Service contribute to achieving the SDGs. 62% affirm the University OHS Service participate in sustainability working groups, although only 48.3% of participants believed that OHS technical or health personnel form part of them.

Based on the results of the survey, an inventory of OHS actions and initiatives were made, classifying them according to the corresponding SDGs. First, the actions identified by the participating universities were reviewed and, where appropriate, reformulated to create a block of 60 “specific actions”; that is, a set of specific tasks undertaken to achieve a specific objective of the OHS departments. These included, for example, the certification of Cardiac Protective Areas or the development of emergency response plans and measures in university facilities. Other specific actions were incorporated into this block, which, while not currently being executed, may be considered effective SDG-related OHS initiatives in Spanish universities.

### 3.2 Phase 2: general actions

The “specific actions” described above were grouped into 9 “general actions” associated with certain OHS initiatives. Thus, all “specific actions” identified in the survey are classified by subject or themes, allowing them to be identified and understood in terms of their impact on the SDGs.

- Certification and accreditation.
- Culture of Health and Safety.
- Emergencies.
- Risk assessment.
- Training, information and awareness.
- Management of emerging risks.
- Health promotion and monitoring.
- Specific protection for workers particularly at risk.
- Monitoring and controlling OHS actions.

### 3.3 Phase 3: dimensions

The general actions listed above are also grouped into the following 3 dimensions.

#### 3.3.1 Person (P)

Actions that seek to safeguard people’s health and well-being through the promotion of safe and healthy habits, creating a positive environment for work and study at the university. This includes, but is not limited to, OHS actions and health and well-being campaigns, such as risk assessments, health promotion campaigns, road safety, training, Cardiac Protected Areas, protection equipment, emergency protocols, equality, etc.

#### 3.3.2 Environment (E)

Actions that contribute to making university campuses and their surrounding areas safe and healthy places and actions that care for the environment. This includes actions related to waste management, disposal of hazardous materials, environmental risk assessments, mobility, OHS design (construction, renovations, PPE), emergency protocols, etc.

#### 3.3.3 Preventive and sustainable culture (C)

Actions that foster a change in behavior and attitudes within the university community, raising awareness of critical issues and modifying behaviors to have a positive impact on society. This includes audits, certifications, awareness campaigns, collaborative projects and initiatives, research and innovation, etc.

### 3.4 Phase 4: SDG impact matrix

To establish the linkage between the actions of the OHS Services and specific SDGs, a structured qualitative analysis was conducted in three phases. First, the open-ended responses provided by the universities were reviewed, which included detailed descriptions of initiatives developed in the fields of health, safety, prevention, or sustainability. Subsequently, each action was compared against the official targets and descriptors of the 17 SDGs, as defined by the United Nations framework, to identify those goals with which there was a direct or functional correspondence.

**Figure 3:**
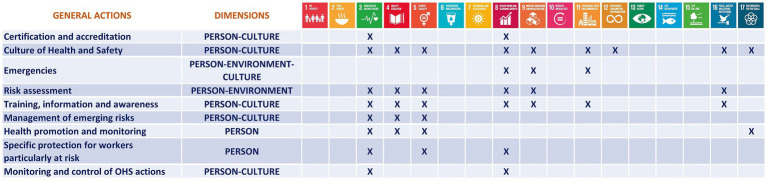
Impact of occupational health and safety (OHS) actions on the SDGs.

For example, the implementation of emergency plans or the designation of cardiac-protected areas was associated with SDG 3 (Good Health and Well-being), while equality policies and protocols for vulnerable groups were linked to SDG 5 (Gender Equality). The classification was initially carried out independently by two researchers and later cross-checked in group review sessions until consensus was reached. This procedure ensured internal consistency, minimized interpretation bias, and facilitated the traceability of each association, which was ultimately systematized in the impact matrix presented in Figure 3.

This matrix provides a comprehensive visualization of how the nine general OHS actions contribute to specific SDGs across the three defined dimensions (Person, Environment, Culture). For example, actions related to risk assessment, health promotion, and the protection of particularly vulnerable workers are strongly linked to SDG 3 and SDG 8 within the Person dimension. In contrast, initiatives such as waste management, environmental risk assessment, and OHS design directly support SDG 11 and SDG 12 within the Environment dimension.

Additionally, cultural actions, including training, awareness campaigns, certifications, and the promotion of a preventive culture, have broad implications, contributing significantly to SDG 4 (Quality Education), SDG 5 (Gender Equality), SDG 16 (Peace, Justice and Strong Institutions), and SDG 17 (Partnerships for the Goals). This matrix highlights the systemic and transversal nature of OHS services and their potential to influence university sustainability policies from multiple angles.

## 4 Discussion and conclusions

This research has highlighted the significant contribution of university OHS services to achieving the SDGs of the 2030 Agenda. Based on the results obtained, several reflections can be made on the impact of these services and their direct and indirect relationship with various SDGs beyond those traditionally associated, such as SDG 3 (Good Health and Well-being) and SDG 8 (Decent Work and Economic Growth). It is crucial to analyze these interactions within a broader context, including aspects such as environmental sustainability, organizational culture, and the overall well-being of the university community.

While international agencies often limit the role of OHS services to the fulfilment of SDGs 3 and 8, the data obtained in this study reveal that the impact of their actions extends across a wider range of SDGs. This finding reinforces the need for a more holistic approach to health, understood as a state of physical, mental, and social well-being, according to the World Health Organization’s definition. In this way, OHS interventions extend to SDGs such as SDG 5 (Gender Equality), SDG 11 (Sustainable Cities and Communities), SDG 16 (Peace, Justice and Strong Institutions), and SDG 17 (Partnerships for the Goals). This demonstrates that the role of OHS services in universities goes beyond their preventive and risk management functions, becoming active agents of change towards sustainability.

Moreover, the inclusion of OHS personnel in university sustainability working groups, as evidenced in Crue, is crucial for promoting a culture of safety, health, and sustainability. This collaboration enables universities to comprehensively address sustainability challenges, generating a positive impact on the entire university community. However, the study’s results show that only 62% of OHS representatives participate in these groups, and less than half (48.3%) of the technical or health staff are involved, indicating room for improvement in integrating these services into institutional sustainability policies.

Another relevant aspect is the identification of three key dimensions in which OHS actions can be grouped: person, environment, and culture. This categorization provides an effective tool for mapping the contributions of the OHS services to the SDGs, facilitating the understanding of the specific impact of their actions in each area. This distinction is also useful for universities as it enables them to implement more targeted and coordinated strategies that address the various aspects of sustainable development from a health and safety perspective.

Finally, the results also invite reflection on the role of universities as agents of social transformation through OHS. As the literature suggests, universities are ideal spaces for experimenting with sustainable policies and practices and OHS services can act as catalysts for integrating these principles at all institutional levels (5, 17). Thus, they not only contribute to improving working conditions and well-being within the campus but also generate a positive societal impact by fostering a culture of prevention and sustainability.

From a public health and organizational sustainability perspective, the concept of positive social impact associated with OHS services can be understood as the ability of these units to generate structural benefits both within and beyond the institutional environment. In the present study, this impact is evidenced in three dimensions: (1) the direct improvement of the work and study environment through preventive actions (such as emergency plans, health promotion campaigns, workplace adaptations for vulnerable groups, or the certification of cardiac-protected areas); (2) the cultural transformation of the university community through awareness-raising activities, training, and active participation in sustainability policies; and (3) the alignment of these actions with the SDGs, linking local interventions to global goals related to human development, equality, decent work, and well-being (SDGs 3, 5, 8, 11, and 17).

Although the study’s methodological design does not include the collection of quantitative outcome indicators, the approach adopted —based on the systematization of good practices and their classification by impact— allows us to infer a substantive contribution of OHS services to the strengthening of institutional structures and collective well-being. In this sense, positive social impact should not be limited to clinical or economic metrics but should also be understood from a holistic perspective that values the transformative effects of preventive policies in complex environments such as universities.

This study complements internationally recognized evaluation frameworks for assessing SDG performance in higher education, such as the Times Higher Education (THE) Impact Rankings. This ranking system assesses universities’ commitment to the SDGs through standardized indicators that include institutional policies, scientific output, collaboration, and community outreach activities. However, these frameworks rarely consider the explicit role of OHS services as active agents of sustainability.

It is noteworthy that 17 of the 29 universities (58.6%) participating in this study are included in the Times Higher Education Impact Rankings 2024, which reinforces the representativeness and relevance of the sample analysed. This overlap allows us to establish a significant connection between the actions of OHS services and the broader institutional efforts aimed at achieving the SDGs. Although such actions are not always explicitly captured by the indicators considered in international rankings, the results suggest that OHS services may be making a substantial—albeit indirect—contribution to the overall sustainability performance of universities.

This observation highlights the importance of considering OHS services in future evaluation frameworks as an integral part of institutional sustainability strategies and reinforces the need to expand current indicators to include metrics that reflect the preventive, educational, and cultural impact of these services within the university environment.

In conclusion, this research has demonstrated that the activities of the universities OHS services have a significant impact on achieving the SDGs and contribute to fulfilling the 2030 Agenda.

More specifically, the following conclusions can be drawn from the study:

International agencies merely recognize SDG 3 and 8 as linked to OHS. We described that the role of OHS services is most clearly recognized in working to create a safe and healthy workplace environment. However, the OHS services do much more than this and the impact of their actions goes beyond these two SDGs.

Taking a broader view, and understanding health as the “state of complete physical, mental and social well-being, and not only the absence of affections or diseases,” we found additional connections with the SDGs, including environmental sustainability and protection. Thus, the Crue report described in this study also incorporated SDG 5, 11, 16 and 17.

In addition, to the list of SDGs identified by the Crue Sustainability Area, SDGs 4, 9 and 12 must be added due to the number of OHS initiatives they group together.

**Figure 4:**
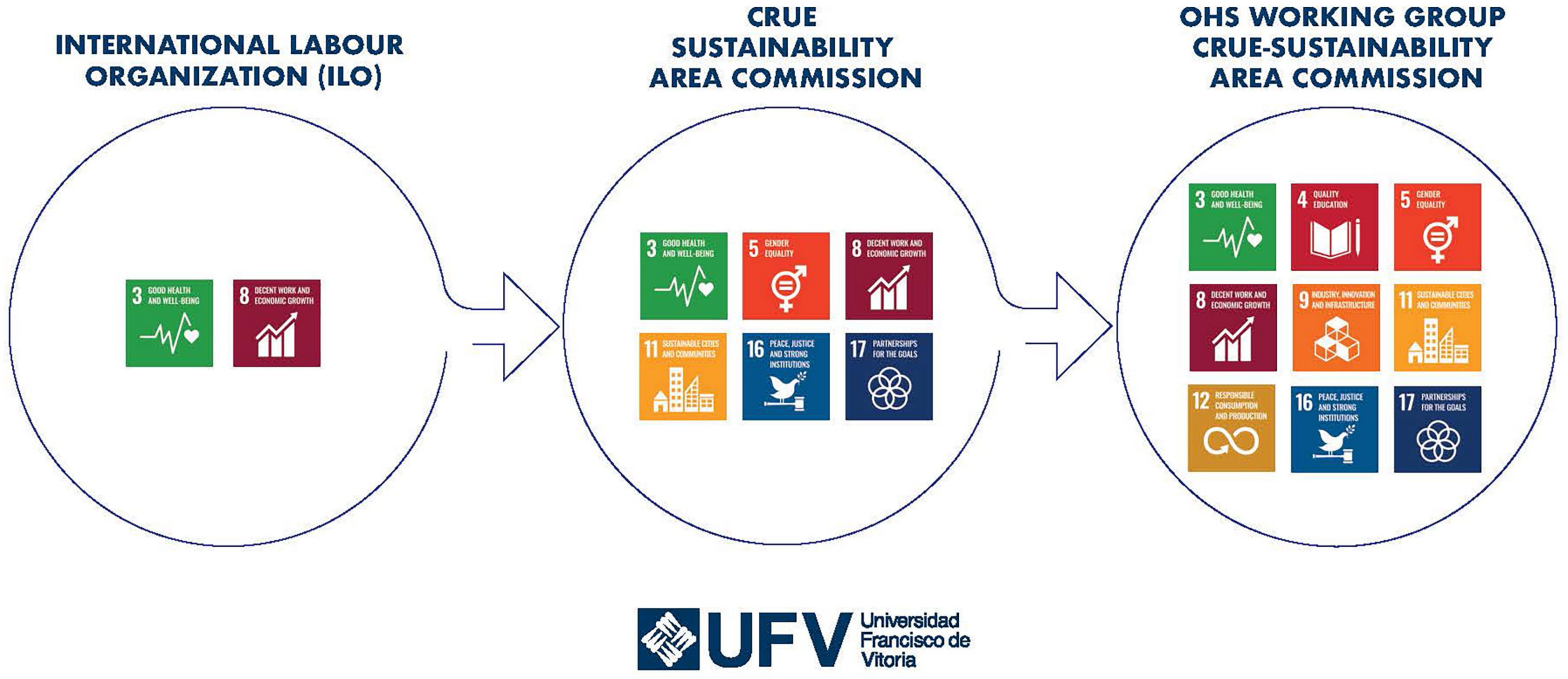
Contributions of occupational health and safety services to SDGs.

Figure 4 offers a visual synthesis of this progression, illustrating how the scope of SDGs associated with OHS services has evolved – from the limited focus on SDG 3 and 8 by the ILO, to the broader inclusion of additional goals (SDG 5, 11, 16, and 17) by the Crue-Sustainability Area Commission, and finally to the expanded set (including SDG 4, 9, and 12) identified through the fieldwork in this study. This layered representation underscores the growing recognition of the multidimensional contribution of OHS services within the framework of the 2030 Agenda.

It is essential that the sustainability working groups of each university incorporate expert OHS staff who can contribute to creating and maintaining a culture of health and safety as well as sustainability in Spanish universities, positively influencing the entire university community. In short, universities must incorporate OHS services into their sustainability committees to enhance their effectiveness in the different areas where their actions have an impact.

The 3 dimensions defined in this study allow for the quick and effective identification of the areas impacted by OHS actions (person, environment, or culture) and relating them to specific SDGs.

Finally, this work has not only identified the SDGs aligned with the OHS services, but also highlighted the impact and contribution these actions can make in society. The SDGs afford the OHS services the opportunity to boost their profile and consolidate their institutional position and importance in fulfilling the 2030 Agenda.

## 5 Limitations and future research

Analysis in this research tends to be entirely descriptive and lacks a more empirical approach. The study focuses on frequency analysis and the qualitative classification of institutional actions linked to the SDGs. This choice responds to the exploratory nature of the work, which aimed to identify good practices and generate an initial structured intervention matrix of OHS services within the Spanish university context.

Thus, a significant limitation of the present study is the absence of comparative analyses between institutional groups, such as public versus private universities, or comparisons based on size, geographical region, or level of engagement in sustainability policies. Although this decision is consistent with the exploratory nature of the study and the aim of providing a general overview of the actions of OHS Services within the Spanish university system, future research should incorporate more detailed segmentations that could reveal differences in the intensity, diversity, or focus of the reported actions.

Furthermore, the database generated —due to its level of detail and structure— offers a suitable starting point for the development of both descriptive and inferential comparative analyses, which could help to characterize different institutional profiles of commitment to the SDGs from the perspective of occupational health and safety.

Future research could enhance this approach by incorporating more robust comparative analyses, such as chi-square tests to detect significant differences between universities based on their type (public or private), size, or level of engagement in sustainability policies, or through clustering techniques that may identify differentiated institutional profiles. This empirical development would not only be useful to validate the hypotheses proposed in this study but also to inform evidence-based university policies within the framework of the 2030 Agenda.

## Data Availability

The raw data supporting the conclusions of this article will be made available by the authors, without undue reservation.
