# Supplementary material for: Contribution of universities health and safety services in achieving the sustainable development goals
Source: Front Public Health. 2025 Jul 29;13:1604003. doi: 10.3389/fpubh.2025.1604003 (PMC12339436; doi:10.3389/fpubh.2025.1604003)
Supplement: Supplementary file 1 [file Table_1.DOCX]

Supplementary Material

# Annex I. Questionnaire of the study

Data collection for the preparation of a guide to align the Occupational Health and Safety Service activities of Spanish Universities with their SDGs.

Data identifying the University participating in the survey will not appear in the results.

1. University:
2. E-mail:
3. Does the Occupational Health and Safety Service participate in specific working groups related to the SDGs of the 2030 Agenda at the University? YES/NO
4. Do officers or medical staff of the Occupational Health and Safety Service participate in these working groups? YES/NO
5. In your university, which SDGs do you identify as OHS actions or initiatives?

|  | I do not know |  | SDG 9 INDUSTRY, INNOVATION AND INFRASTRUCTURE: Build resilient infrastructure, promote inclusive and sustainable industrialisation and foster innovation |
| --- | --- | --- | --- |
|  | SDG 1 NO POVERTY: End poverty in all its forms everywhere. |  | SDG 10 REDUCED INEQUALITIES: Reduce inequality within and among countries. |
|  | SDG 2 ZERO HUNGER: End hunger, achieve food security and improved nutrition, promote sustainable agriculture. |  | SDG 11 SUSTAINABLE CITIES AND COMMUNITIES: Make cities and human settlements inclusive, safe, resilient and sustainable. |
|  | SDG 3 GOODHEALTH AND WELL-BEING: Ensure healthy lives and promote well-being for all at all ages. |  | SDG 12 RESPONSIBLE CONSUMPTION AND PRODUCTION: Ensure sustainable consumption and production patterns. |
|  | SDG 4 QUALITY EDUCATION: Ensuring inclusive and equitable quality education and promote lifelong learning opportunities for all. |  | SDG 13 CLIMATE ACTION: Take urgent action to combat climate change and its impacts. |
|  | SDG 5 GENDER EQUALITY: Achieve gender equality, empower all women and girls. |  | SDG 14 LIFE BELOW WATER: Conserve and sustainably use the oceans, seas and marine resources for sustainable development. |
|  | SDG 6 CLEAN WATER AND SANITATION: Ensure the availability and sustainable management of water and sanitation for all. |  | SDG 15 LIFE ON LAND: Protect, restore and promote sustainable use of terrestrial ecosystems, sustainably manage forests, combat desertification, and halt and reverse land degradation and halt biodiversity loss. |
|  | SDG 7 AFFORDABLE AND CLEAN ENERGY: Ensure access to affordable, reliable, sustainable and modern energy for all. |  | SDG 16 PEACE, JUSTICE AND STRONG INSTITUTIONS: Promote peaceful and inclusive societies for sustainable development, provide access to justice for all and build effective, accountable and inclusive institutions at all levels. |
|  | SDG 8 DECENT WORK AND ECONOMIC GROWTH: Promote sustained, inclusive and sustainable economic growth, full and productive employment and decent work for all. |  | SDG 17 PARTNERSHIPS FOR THE GOALS: Strengthen the means of implementation and revitalise the Global Partnership for Sustainable Development |

1. Briefly detail, according to the SDGs identified in the previous question, the actions or initiatives developed. Please list these activities by SDG.
2. Do you think that the preventive activities developed by the Prevention Services could contribute to achieving the SDGs? YES/NO
3. If you would like to propose OHS initiatives to achieve the SDGs in Spanish Universities, please indicate below:
4. Basic information regarding the protection of your personal data: the person responsible for the processing of your data is the Universidad Francisco de Vitoria (UFV).

The purpose of the data processing is the preparation of a guide to align the activities of the Prevention Services of Spanish Universities with the SDGs, as well as the sending, by any means, including electronic means (by way of example, but not limited to: email), of those communications that are necessary, where appropriate, to expand or clarify the information collected. The data provided through this form must be truthful and current and correspond to the identity of the party interested in the event that personal data are provided. The legitimacy of the processing is the consent of the data subject by ticking the boxes provided for this purpose. The information provided will not be disclosed to third parties, except to service providers with access to data. Personal data (contact e-mail address) will be retained for the duration of the processing of the request or administrative task.

You may exercise your rights of access, rectification, suppression, opposition, limitation of processing and portability by writing to the General Secretariat of the Universidad Francisco de Vitoria, at the following address: Carretera M-515 de Pozuelo a Majadahonda, Km. 1,800, 28223 Pozuelo de Alarcón (Madrid), or by e-mail to dpd@ufv.es (mailto: dpd@ufv.es).

If you provide data of third parties through this form, we inform you that you are obliged to inform the interested party of the content of this clause. Further information can be found on the website: https://www.ufv.es/politica-de-privacidad/*

I accept and authorise the Universidad Francisco de Vitoria to process my data for the aforementioned purposes and to send me, by any means, including electronic means (by way of example, but not limited to: e-mail), such communications as may be necessary, where appropriate, to extend or clarify the information collected.
